# The association between changes in functional capacity and work ability among unemployed individuals

**DOI:** 10.1007/s00420-019-01498-1

**Published:** 2019-12-14

**Authors:** Minna Savinainen, Jorma Seitsamo, Matti Joensuu

**Affiliations:** 1 Finnish Institute of Occupational Health, 33540 Tampere, Finland; 2 Finnish Institute of Occupational Health, 00290 Helsinki, Finland

**Keywords:** Work ability, Functioning, Health, Unemployment, Longitudinal study

## Abstract

**Purpose:**

Unemployment has multidimensional effects. This study investigated how the changes in functioning are associated with the changes in perceived work ability among unemployed people.

**Methods:**

The participants were clients in projects funded by the European Social Fund (ESF) targeted for unemployed individuals. They answered a questionnaire covering work ability (Work Ability Score) and functioning (cognitive, psychological and social functioning, physical condition and everyday activities) and perceived health at the beginning and at the end of the project (mean follow-up 173 days). The study included data from unemployed respondents (*N* = 502) aged 19–64 years.

**Results:**

Overall, during the follow-up, both work ability and different aspects of functioning improved, excluding physical condition. Changes in cognitive and psychological functioning, physical condition and everyday activities were significantly associated with the changes in work ability. The physical condition had the strongest association with the changes in work ability. Short unemployment time and especially good perceived health improved WAS over time. Age, gender and follow-up time were not associated with changes in work ability.

**Conclusions:**

Maintaining or improving health and functioning and shortening the length of unemployment appeared to be important issues in enhancing work ability and thus increasing re-employability potential.

## Introduction

Prolonged unemployment is associated with deteriorating health (e.g. Nurmela et al. 2018; Worach-Kardas and Kostrzewski 2014; Butterworth et al. 2012; Pharr et al. 2012) and work ability (Hult and Lappalainen 2018; Lappalainen et al. 2017; Lundin et al. 2016; Szlachta et al. 2012). The proportions of unemployed persons who perceive themselves as having good work ability (a score of eight or above on a scale of 0–10) vary between 30% and 60% in different studies (e.g. Hult and Lappalainen 2018; Hult et al. 2017; Kerätär et al. 2016; Szlachta et al. 2012). The associations of unemployment and health vary depending on the economic situation of the household, perceived health status, personal relationships, the sense of ability to work, social roles and the individual perception of one’s life position (Brand 2015; Worach-Kardas and Kostrzewski 2014).

Functioning illustrates an individual’s ability to take care of oneself and one’s household, run errands and perform everyday tasks at home, within hobbies, or studies, or at work (Hult and Lappalainen 2018). Functioning is closely related to health, and it can be described through psychological (Neff et al. 2007), social (Brackett et al. 2006), physical (Savinainen et al. 2004) and cognitive (Roberts et al. 2007) dimensions. There are only a few studies on all aspects of functioning and work ability of unemployed persons. Usually, these studies concern relations between work ability (work participation) and health (Ferreira et al. 2015; McGonagle et al. 2015; Kuijer et al. 2012; Hoving et al. 2010) or only some aspects of functional capacity (Hult and Lappalainen 2018; Worach-Kardas and Kostrzewski 2014; Vastamäki et al. 2014; Szlachta et al. 2012; Soer et al. 2012). Higher functioning and physical condition have been found to be more influential in the association with health and work ability than psychosocial factors for long-term unemployed (Hult and Lappalainen 2018). Personal and social resources can help individuals to avoid and cope with harmful stressors. However, these resources decrease during employment (Vastamäki et al. 2014; Szlachta et al. 2012).

Good health is a key resource for unemployed persons (Hult et al. 2017; Ferreira et al. 2015; van de Vijfeijke et al. 2013), but also education, skills, values and attitudes contribute to an individual’s perception of his or her work ability (Hult and Lappalainen 2018; Lappalainen et al. 2017; Vastamäki et al. 2014; Szlachta et al. 2012; Pensola et al. 2008). In addition, successful coping style (McGonagle et al. 2015; Wageneer et al. 2015) and leisure time physical exercise predict higher work ability (van de Vijfeijke et al. 2013; Lindberg et al. 2006). Many studies concerning work ability and functioning among unemployed are restricted to diagnose group (e.g. Hayward et al. 2018; Deckersbach et al. 2016; Sanger et al. 2016).

Maintaining work ability during unemployment is important because it directly affects possibilities for re-employment (McGonagle et al. 2015). Work ability is defined as a balance between personal resources and job demands (Ilmarinen et al. 2008) and therefore dependent on the operational environment (McGonagle et al. 2015). For the unemployed, however, a frame of reference for one’s work ability, namely a current work setting, does not exist, which makes it harder for the individuals and professionals to evaluate work ability directly. Information on functioning has been shown to influence the work ability evaluations physicians make (Wind et al. 2009). Therefore, longitudinal studies are needed to show how functioning and work ability are related and could work ability be raised by enhancing functioning of those unemployed.

The aims of this study were to investigate how the changes in functioning are associated with the changes in perceived work ability among unemployed persons. We also analysed which aspects of functioning are the most relevant concerning changes in work ability and how the length of the unemployment period effects the associations.

## Methods

### Participants

The study population came from the ongoing project ‘Social inclusion and the changes of one’s work ability and capacity (Solmu) coordination’ financed by the European Union. The participants were the clients in projects funded by the European Social Fund (ESF) targeted at unemployed individuals. The projects were aimed at increasing inclusion and improving functional capacity by, e.g. developing services for unemployed, giving individual support and guidance to the participants. The participants were from 21 different projects, and the number of participants per project differed from two to 158. The length and content of the different projects differed a lot. Usually, the development projects took long period (up to 3 years), whereas some projects lasted only a couple of weeks for participants. The national project, which had over 150 participants, developed and established the operation of multidisciplinary joint services to promote employment in six different municipalities. They developed especially new assessment tools for work ability and functioning, group methods for social rehabilitation and health examination model for unemployed. These actions were indirectly supposed to influence work ability and functioning in a long term.

Inclusion criteria for the participants were that the participant was unemployed, had earlier employment history, had answered the study questionnaire twice, and the follow-up time was 30 days or over. The mean follow-up time was 173 days (31–780 days, SD 128). Questionnaires concerning functioning and work ability were completed both at the beginning and at the end of the project, either independently by the participants themselves or together with a project employee. The background variables were only asked at the beginning, i.e. age, gender, basic and occupational education, length of unemployment and employment status. By the end of April 2018, altogether 502 persons across Finland were included in the study. Informed consent was obtained from all individual participants included in the study.

### Measures

#### Work ability

Work ability was measured by the Work Ability Score (WAS), a first item of the Work Ability Index developed in the early 1980s by the Finnish Institute of Occupational Health (Tuomi et al. 1998). WAS has proved to be a reliable indicator of work ability in many studies (i.e. Leijten et al. 2014; von Bonsdorff et al. 2011). Participants were asked the following: ‘Assuming that your best work ability would receive a score of 10 points, how many points would you give your present work ability?; If you do not currently work, give your assessment in relation to your last job, or the demands of your occupation’. The scale ranged between 0 (not able to work at all) and 10 (work ability at its best).

#### Independent factors

For the purposes of this study, several summative scales were created. Psychological functioning was measured with the Warwich-Edinburgh Mental Well-Being Scale (WEMWBS) (Tennant et al. 2007). It covers items of feeling hopeful about the future, relaxed, useful, able to solve problems, clear thinking, closeness to others and being able to make own decisions (1 = never, 2 = seldom, 3 = sometimes, 4 = often, 5 = all the time) and showed good internal consistency (Cronbach’s alpha 0.83).

Cognitive functioning (Cronbach’s alpha 0.74) consisted of three items: Can you normally concentrate on things, are you able to take in new knowledge and learn new skills (1 = very well, 2 = well, 3 = satisfactory, 4 = poor, 5 = very poor) and how do you currently rate your memory (1 = very poor, 2 = poor, 3 = satisfactory, 4 = good, 5 = very good)? (von Bonsdorff et al. 2011).

Social functioning (Cronbach’s alpha 0.74) was assessed with four items: How well do the following statements describe your situation? Choose a number on a scale of 1 = completely disagree to 5 = completely agree: I get on well with those close to me; I find it easy to maintain my friendships; I find it easy to get to know new people; I find it easy to socialize with people I do not know.

Physical condition was enquired with the question ‘In your opinion, what is your level of physical fitness?’ based on the scale: 1 = good, 2 = fairly good, 3 = average, 4 = fairly poor, 5 = poor (Aromaa and Koskinen 2002).

Personal abilities to cope with everyday tasks (everyday activities) were enquired and covered housework, using public services, getting around outside home, and using the Internet (1 = I cope well, 2 = I have some trouble coping, 3 = I have a lot of trouble coping, 4 = I am unable to cope). The items were dichotomized as 1 = a person copes well, and 0 = a person has at least some difficulties. Further, the responses were summed up indicating the number of difficulties (range 0–4).

All the above summative measures of functioning were transformed to a 0 to 10 scale with a higher score indicating better functioning.

Perceived health was assessed with the question: In your opinion, is your health currently’ 5 = very good, 4 = fairly good, 3 = average, 2 = fairly poor, or 1 = poor? (Bowling 2005).

Age, length of unemployment, and length of follow-up time in months were also included in this study. The length of unemployment was dichotomized as two categories: less than three years and three years or over. These categories were based on the study results by Janlert et al. (2015), where the incidence of deteriorated health and health behaviour increased after three years of unemployment.

### Statistical analysis

Baseline characteristics were presented as frequency tables and means. Differences between gender and length of unemployment were tested with Chi-square statistics and *t* tests. The correlations were tested with Spearman’s rho coefficients (*r*_s_). These statistical analyses were performed using SPSS 25 software.

Linear regression analyses were performed to study the differences in work ability and domains of functioning using only information from baseline data, adjusted for age, sex, vocational education, length of unemployment (less than 3 years vs 3 years or more), and perceived health (classified as good, average, and poor health).

The associations between functional capacity, self-rated health and background variables were assessed by the generalized estimating equations (GEE) technique, which considers that the repeated measurements are correlated within the respondents. This likelihood-based technique is valid under missing at random assumption when missing data might depend on observed data (Diggle et al. 2002). With this method, it was possible to use all available data from both time points and there was no need to be restricted to the endpoint data only. The obtained regression coefficients are pooled coefficients of within-subject and between-subject longitudinal relationships, that is, the magnitude of the coefficients indicates both the change over time and differences between the respondents (Twisk 2004). The independent variables were analysed as time-dependent variables on a 0 (poor) to 10 (excellent) scale, except for age, sex, vocational education, and length of unemployment. The analyses were carried out in three phases. First, the effects of the different dimensions of functional capacity, on the WAS, were analysed separately (Model 1). The analysis was adjusted with age, gender, and vocational education. Second, length of unemployment (less than 3 years vs 3 years or more) and perceived health (classified as good, average, and poor health) were included in the model (Model 2). Finally, a multivariate model was carried out. This model included all the aspects of functional capacity and other predictors used in the previous models. At this phase, all the first-order interactions were tested. The significance level used for all tests was *p* ≤ 0.05. The GEE-analyses were performed using SAS 9.4 software.

## Results

The average age of the participants was 42.2 years (19–64 years; SD 12.9). The proportion of men and women was almost even. Over 40% of the participants perceived their health at least fairly good, and almost 70% reported some longitudinal diseases/symptoms or impairment. Almost everyone (99%) had completed comprehensive school, and over half of the population (56%) had at least a vocational education. About 60% of the study population had been unemployed 3 years or over. At the baseline, all the participants were unemployed, but in addition half of the participants were involved in practical training, workshop, work trial, or rehabilitative work activity, six percentage had sick leave, and seven percentage had something else, e.g. were homemaker or caring for close relative. There were no statistically significant differences between gender in age, educational background, and length of employment. Instead, there was a statistically significant difference between gender in reported longitudinal disease/symptom or impairment. The men reported less diseases than women also at the beginning (62.5% vs. 73.4%, respectively, *p* = 0.010) and at the end of the follow-up (61.8% vs 72.0%, respectively, *p* = 0.018). Additionally, there were statistically significant differences in education between groups with different lengths of unemployment. Among individuals who have been unemployed 3 years or over, it was more common to have no education after comprehensive school (49.7% vs. 36.0%, respectively, *p* = 0.007) (Table 1).

**Table 1:** Background information of the study participants in the beginning of the study (*N* = 502) and in the different groups based on the length of unemployment

Variable	All (%)	Length of unemployment under 3 years (%)	Length of unemployment 3 years or over (%)
Gender			
Male	50.5	49.5	51.2
Female	49.3	50.0	48.8
Other	0.2	0.5	0
Perceived health			
Good	12.8	14.5	11.6
Fairly good	29.7	33.0	27.6
Average	36.5	37.0	36.2
Fairly poor	17.8	14.5	19.9
Poor	3.2	1.0	4.7
Post-comprehensive education			
No education after comprehensive school	44.2	36.0	49.7
Vocational school	49.0	57.5	43.4
Bachelor degree or higher	6.8	6.5	7.0

Overall, during the follow-up, both work ability and different aspects of functioning were improved, except for physical condition (Table 2). There were no statistically significant differences in work ability between genders. However, in the beginning, there were statistically significant differences between gender in psychological functioning and physical condition. Women had better psychological functioning than men (7.6 vs 7.3, *p* = 0.013), whereas men had a better physical condition than women (5.2 vs. 4.9, *p* = 0.044). At the end of the study, there was a statistically significant difference between gender only in psychological functioning; women still had better psychological functioning than men (7.9 vs. 7.3, *p* = 0.001).

**Table 2:** Basic results of the work ability and the different aspects of functional capacity in the beginning and at the end of the follow-up (scale 0–10; mean, SD) among all the participants and in groups with different lengths of unemployment (under 3 years and 3 years or over)

Variable (*n*)	In the beginning Mean (SD)	At the end of the follow-up Mean (SD)
Work Ability Score (*n* = 495)	6.2 (2.4)	6.5 (2.3)
Length of unemployment under 3 years (*n* = 197)	6.5 (2.3)	6.7 (2.3)
Length of unemployment 3 years or over (*n* = 298)	5.9 (2.5)	6.3 (2.3)
Psychological functioning (*n* = 497)	7.5 (1.8)	7.6 (1.8)
Length of unemployment under 3 years (*n* = 199)	7.4 (1.8)	7.6 (1.8)
Length of unemployment 3 years or over (*n* = 298)	7.5 (1.7)	7.6 (1.8)
Cognitive functioning (*n* = 495)	7.5 (2.2)	7.8 (2.0)
Length of unemployment under 3 years (*n* = 200)	7.5 (2.3)	7.8 (2.0)
Length of unemployment 3 years or over (*n* = 295)	7.6 (2.1)	7.8 (2.1)
Social functioning (*n* = 470)	8.5 (2.0)	8.6 (1.9)
Length of unemployment under 3 years (*n* = 179)	8.5 (2.1)	8.7 (1.8)
Length of unemployment 3 years or over (*n* = 291)	8.6 (2.0)	8.6 (2.0)
Physical condition (*n* = 493)	5.1 (2.7)	5.1 (2.7)
Length of unemployment under 3 years (*n* = 199)	5.3 (2.6)	5.1 (2.6)
Length of unemployment 3 years or over (*n* = 294)	4.9 (2.8)	5.0 (2.7)
Everyday activities (*n* = 500)	5.6. (3.4)	5.8 (3.4)
Length of unemployment under 3 years (*n* = 200)	5.7 (3.6)	5.9 (3.4)
Length of unemployment 3 years or over (*n* = 300)	5.6 (3.3)	5.7 (3.4)

There was a statistically significant difference between the groups with different lengths of unemployment only in work ability. Those who had been unemployed under 3 years had better work ability in the beginning than those who had been unemployed 3 years or over (6.5 vs. 5.9, *p* = 0.007).

The results of the baseline regression analyses are presented in Table 3. Except social functioning, all aspects of functioning had a significant impact on WAS. Physical condition had the strongest positive effect (0.20, *p* < 0.001).

**Table 3:** Linear regression analyses of determinants of WAS (0–10 point scale) at baseline among unemployed men and women (*N* = 478). Parameter estimates (Est) and 95% confidence intervals (CI)

	Est	95% CI
Psychological functioning	0.13	0.04–0.22^**^
Cognitive functioning	0.11	0.04–0.18^**^
Social functioning	0.02	− 0.05–0.08^ns^
Physical condition	0.20	0.03–0.14^***^
Everyday activities	0.12	0.07–0.16^***^

All determinants were adjusted for age, time, gender, vocational education, length of unemployment, and perceived health

*ns* not statistically significant

*p < 0.05

**p < 0.01

***p < 0.001

The analysis adjusted for time, age, gender, and vocational education showed that the effects of each aspect of functioning were highly associated with WAS (Table 4). The highest estimate was in psychological functioning (Est 0.63, 95% CI 0.53–0.72), indicating that a one-unit increase on average in psychological functioning was associated with a 0.63 increase in WAS (Table 4, Model 1). The effects were weaker when perceived health and the length of unemployment were added into the model. However, the effects were still statistically very significant (Table 4, Model 2).

**Table 4:** Predictors of WAS including aspects of functioning and background variables (Model 1) and added with the length of unemployment and perceived health (Model 2). Univariate models over the 3- to 24-month follow-up among unemployed men and women (*N* = 478). GEE estimates (Est) and 95% confidence intervals (CI)

	Model 1		Model 2	
	Est	95% CI	Est	95% CI
Psychological functioning	0.63	0.53–0.72^***^	0.33	0.24–0.41^***^
Cognitive functioning	0.53	0.46–0.61^***^	0.28	0.20–0.35^***^
Social functioning	0.42	0.32–0.51^***^	0.21	0.13–0.29^***^
Physical condition	0.49	0.44–0.54^***^	0.26	0.20–0.31^***^
Everyday activities	0.32	0.27–0.36^***^	0.15	0.11–0.20^***^

Model 1: All predictors were separately adjusted for age, time, gender, and vocational education

Model 2: Model 1 + length of unemployment and perceived health

*ns* not statistically significant

*p < 0.05

**p < 0.01

***p < 0.001

The results of the multivariate model (Table 5) showed that short unemployment time and especially good perceived health improved WAS over time. However, psychological functioning, cognitive functioning, physical condition, and everyday activities all had a significant effect on work ability. Physical condition had the strongest effect, indicating that a 1-unit increase in physical condition was associated with a 0.19-unit increase in the WAS. The effect of social functioning was not statistically significant. Time had only a marginal effect on WAS in all models. To clarify the within-subject changes over time, all the first-order interactions between aspects of functioning and time were tested. Except for physical condition, none of these were statistically significant (Table 5). The negative value of the estimate (− 0.09) indicates that the positive effect of physical condition on WAS decreases slightly over time.

**Table 5:** GEE estimates (Est) and confidence intervals (CI) for WAS between the length of unemployment and changes in aspects of functioning and perceived health, and interactions (×) between functioning and changes over time. A multivariate model over 3- to 24-month follow-up among unemployed men and women (*N* = 478)

	Est	95% CI
Psychological functioning	0.12	0.04–0.20^**^
Cognitive functioning	0.16	0.09–0.22^***^
Social functioning	0.07	− 0.01–0.13 ^ns^
Physical condition	0.19	0.14–0.24^***^
Everyday activities	0.06	0.02–0.10^**^
Length of unemployment		
3 years or more	0	
Less than 3 years	0.54	0.27–0.81^***^
Self-rated health		
Good	1.90	1.49–2.32^***^
Moderate	1.15	0.81–1.50^***^
Poor	0	
Psychological functioning × time	− 0.03	− 0.15–0.09^ns^
Cognitive functioning × time	− 0.03	− 0.13–0.07^ns^
Social functioning × time	0.08	− 0.01–0.16^ns^
Physical condition × time	− 0.09	− 0.16 to − 0.03^*^
Everyday activities × time	− 0.01	− 0.06–0.04^ns^

All predictors, background factors, length of unemployment, and perceived health

*ns* not statistically significant

*p < 0.05

**p < 0.01

***p < 0.001

## Discussion

In this study, the changes in different aspects of functional capacity and the changes in work ability of unemployed people were investigated, along with self-rated health and length of unemployment. The main result of our study was that different aspects of functioning, other than social functioning, had a significant effect on work ability. Physical condition had the strongest effect. The result concerning the role of physical condition with respect to work ability was parallel with earlier study results (e.g. Hult and Lappalainen 2018). In our study, the perceived physical functioning was quite low; the mean was only 5.1 on a 0–10 scale. In addition, it did not improve during the follow-up. Although the aspects of functioning were related to changes in work ability, the effects became weaker as perceived health and the length of unemployment were included in the model.

The significance of health in assessing perceived work ability has been noted in earlier studies (Hult et al. 2017; Ferreira et al. 2015; McGonagle et al. 2015; van de Vijfeijke et al. 2013; Kuijer et al. 2012; Ahlstrom et al. 2010). Our results were concurrent with the results of these earlier studies. Ahlstrom et al. (2010) have also demonstrated high correlations between the WAS and self-reported symptoms and health. In addition, Szlachta et al. (2012) noticed that people tend to regard themselves as able to work by evaluating the condition of their health. Based on the study results by Pharr et al. (2012), unemployed persons had significantly worse perceived mental health profiles, and they were more likely to delay healthcare services due to cost and were less likely to have access to health care than employed persons and individuals voluntarily out of the labour force.

Diverse personal and social resources help the individual cope with and avoid potentially harmful stressors. However, these resources usually decrease during unemployment (Vastamäki et al. 2014; Szlachta et al. 2012). The study by Hult and Lappalainen (2018) found that maintaining personal relationships was associated with good work ability. Nevertheless, in our study, social functioning was not associated with changes in work ability. One reason for that might be that participants’ social functioning was quite high already at the beginning of the study; the mean was 8.5 on a 0–10 scale.

At baseline, the mean work ability was 6.2 and over 42% of the study population perceived health at least as fairly good. Our result concerning work ability among unemployed persons was a little bit lower than in the study by Szlachta et al. (2012) (6.2 vs. 6.8, respectively). There were no differences between gender in work ability and self-rated health. The work ability results are parallel with the study by Padula et al. (2012) but deviated from an earlier study by Worach-Kardas and Kostrzewski (2014) where women reported chronic disorders more often than men.

Work ability was lower among participants who had been unemployed 3 years or over. According to earlier cross-sectional studies, work ability declined when unemployment was prolonged (Hult and Lappalainen 2018; Lappalainen et al. 2017; Lundin et al. 2016; Szlachta et al. 2012) and our findings confirm that. Additionally, Szlachta et al. (2012) found that repeated failures in gaining employment, deteriorating well-being, and lowered self-esteem, are associated with a longer period of unemployment. There were no differences in work ability at the end of the follow-up between the groups with different lengths of unemployment. Our results are very encouraging and suggest that different interventions are worthwhile among unemployed persons. One reason for our results might be that study participants participated in projects whose targets were, e.g. to increase inclusion and improve functioning, although the results by van Rijn et al. (2016) review study confirmed no evidence for any effect of re-employment programmes on functioning and mental health. The actions of these projects were indirectly supposed to influence work ability in long term, e.g. with more fluent services and processes. The other reason might be a selection effect: those who had higher functioning did not retire from the project in which they participated.

Over 90% of the participants had no education after comprehensive school or had completed vocational education. The results of this study probably reflect the nature of the participants’ previous jobs and occupations, characterized by physical strain, which leads to an emphasis on health and physical functioning in their perceptions of their work ability. Most people, however, have a reasonable degree of work ability during most of their professional lives, even if most of us will, from time to time, temporarily suffer from reduced work ability (e.g. due to ill health). There is also (at any specific time) a relatively small group of people that have work ability, but who are unemployed for some reasons, e.g. due to scarcity of work. Finally, there is a minority that permanently have no, or reduced, work ability, due to dysfunctions or chronic illness (Tengland 2011).

According to Lundin et al. (2016) self-assessed poor work ability seems to be an indicator of future labour market exclusion of different kinds and can be used in public health monitoring. In earlier studies, self-assessed poor ability has predicted future long-term sickness absence, disability pension (Bethge et al. 2018; Jääskeläinen et al. 2016), and long-term unemployment. For example, in the Kerätär et al. (2016) study concerning unemployed citizens, 27% were found unable to work in the open labour market, care or rehabilitation was seen as necessary to enable return to work for 20%, and 15% were eligible for a disability pension. The predictability of work ability could be used as a guide in tailoring interventions and rehabilitation activities (Bethge et al. 2018). The single-item question on work ability could be used as a simple indicator for assessing the status and progress of work ability (Ahlstrom et al. 2010).

A comprehensive work ability assessment and the unemployed person’s own opinion of his or her work ability are important (Hult and Lappalainen, 2018). Assessing the work ability of a jobseeker is challenging. Some have never had paid work allowing for comparison of their abilities. Moreover, the participants did not necessarily self-recognize their disabling impairments (Kerätär et al. 2016). Therefore, more focus should be placed on finding employment based on existing skills and remaining work ability. Many unemployed people with a long-term illness would be fit for work if they received proper treatment for their illness and possible restrictions would be considered in outlining their duties at work (Hult and Lappalainen 2018). A notable proportion of instances of poor work ability among the long-term unemployed is not detected by ordinary health services. This indicates weakness at the point of contact to the service system. Reasons for this can include both the cost of care and the structures of health services (Nurmela et al. 2018).

Novel ways to assess work and functional capacity among the unemployed should be implemented in the health and employment services. When we can determine aspects of functional capacity which influence changes of work ability, we may be able to better promote those aspects of functional capacity (e.g. appropriate support or intervention) which increase work ability and promote re-employability. The re-employment of the long-term unemployed person increases the person’s own financial situation and decreases the labour market subsidy by the municipality and state, which ultimately has a positive effect on society as a hole.

To our knowledge, it is less frequent to study the associations between changes in different aspects of functional capacity and work ability among unemployed persons. The nonsignificant within-subject effects of functioning to work ability suggest that the individual differences did not change over time. Improvement in work ability was obvious whether it was poor or good at the beginning of the study. In clinical studies, work participation has not been included as an outcome measure. More often the focus of research is on the effectiveness of different interventions (Audhoe et al. 2010), rehabilitation in special occupations or age groups, or within specific diagnoses or morbidity (Achterberg et al. 2009).

Our study group consisted of long-term unemployed people, a group that tends to be underrepresented in population-based studies. Collection of the data was performed across a wide area in Finland, which makes it possible to generalize the results nationally. We used generalized estimation equations analyses in this study. Using this technique, it was possible to include in the analysis all the variations in independent and dependent factors at both time points (Diggle et al. 2002). The advantages of this method are obvious: in a typical study setting the endpoint factor is explained by the baseline variables, and the changes in variables between the follow-up period are lost.

### Limitations

The data in this study were based on questionnaire results which could mean that there was also a possibility of bias resulting from self-reported information. The participants may have over- or under-reported information if they perceived it to be a socially desirable response. A longitudinal study design was used without a randomization or comparison group; having such a group could have strengthened the results. The participants were involved in 21 different kinds of projects and interventions with different follow-up time. Overall, the work ability and functional capacity of the participants improved during the interventions. As a further study topic, it could be interesting to detect which kinds of interventions are the most effective for improving functional capacity and how long the results are maintained.

## Conclusion

Maintaining or improving good health and functioning and shortening the length of unemployment appeared to be important issues in maintaining work ability and thus increasing re-employability potential. Consequently, there are needs for assessment and treatment for health and functioning among long-term unemployed people. To identify these people, close cooperation between employment services and health services is essential.
